# Localisation and Mislocalisation of the Interferon-Inducible Immunity-Related GTPase, Irgm1 (LRG-47) in Mouse Cells

**DOI:** 10.1371/journal.pone.0008648

**Published:** 2010-01-13

**Authors:** Yang O. Zhao, Stephanie Könen-Waisman, Gregory A. Taylor, Sascha Martens, Jonathan C. Howard

**Affiliations:** 1 Institute for Genetics, University of Cologne, Cologne, Germany; 2 Departments of Medicine, Molecular Genetics and Microbiology, and Immunology, Division of Geriatrics and Center for the Study of Aging and Human Development, Duke University Medical Center, Durham, North Carolina, United States of America; 3 Geriatric Research, Education, and Clinical Center, VA Medical Center, Durham, North Carolina, United States of America; University of California Merced, United States of America

## Abstract

Irgm1 (LRG-47) is an interferon-inducible Golgi membrane associated GTPase of the mouse whose disruption causes susceptibility to many different intracellular pathogens. Irgm1 has been variously interpreted as a regulator of homologous effector GTPases of the IRG family, a regulator of phagosome maturation and as an initiator of autophagy in interferon-induced cells. We find that endogenous Irgm1 localises to late endosomal and lysosomal compartments in addition to the Golgi membranes. The targeting motif known to be required for Golgi localisation is surprisingly also required for endolysosomal localisation. However, unlike Golgi localisation, localisation to the endolysosomal system also requires the functional integrity of the nucleotide binding site, and thus probably reflects transient activation. Golgi localisation is lost when Irgm1 is tagged at either N- or C-termini with EGFP, while localisation to the endolysosomal system is relatively favoured. N-terminally tagged Irgm1 localises predominantly to early endosomes, while C-terminally tagged Irgm1 localises to late endosomes and lysosomes. Both these anomalous distributions are reversed by inactivation of the nucleotide binding site, and the tagged proteins both revert to Golgi membrane localisation. Irgm1 is the first IRG protein to be found associated with the endolysosomal membrane system in addition to either Golgi (Irgm1 and Irgm2) or ER (Irgm3) membranes, and we interpret the result to be in favour of a regulatory function of IRGM proteins at cellular membrane systems. In future analyses it should be borne in mind that tagging of Irgm1 leads to loss of Golgi localisation and enhanced localisation on endolysosomal membranes, probably as a result of constitutive activation.

## Introduction

The interferon-inducible immunity-related GTPases (IRG proteins, previously named p47 GTPases) are resistance factors against intracellular pathogens in mice. Irgm1 has been reported to be a uniquely powerful member of the IRG family, because mice deficient in this gene showed complete loss of resistance to all bacterial and protozoal pathogens tested so far (reviewed in [1], [2]). Several other IRG proteins have also been shown to be required for complete resistance to *Toxoplasma gondii* (Irgm3, Irgd, Irgb6) (reviewed in [3]) or *Chlamydia trachomatis* (Irgm3 and Irgb10) [4], [5], but resistance functions against other pathogens have not been documented for these proteins. The resistance mechanisms of IRG proteins are still unclear. In the case of resistance to *T. gondii*, which has been most comprehensively documented, interest is focused on the unexplained ability of IRG proteins to cause disruption of the parasitophorous vacuole, resulting in the death of the parasite in the cytosol [6], [7]. Since, alone of the IRG proteins assayed, Irgm1 has never been found at the parasitophorous vacuole, it is clear that this IRG protein is not directly involved in the observed vesiculation and disruption of the parasitophorous vacuole membrane. It has, however, been shown that Irgm1, with its closest homologues Irgm2 and Irgm3, is required for regulating the GTPase cycle of the other IRG proteins in the cytoplasm [8]. It follows that the general susceptibility of Irgm1-deficient mice to protozoal infection may be accounted for as a regulatory failure that disables the IRG-dependent resistance mechanism. It is also possible that dysregulation of the IRG system in absence of Irgm1 may lead to cytopathic consequences that are manifested as defects in hematopoiesis [9], lymphocyte development and/or homeostasis [10],and macrophage functioning [11]. Other mechanisms have, however, been proposed for the role of Irgm1 in these and other aspects of immunity, especially in the case of resistance to *Mycobacteria*, which is lost in Irgm1 deficiency: these include accelerated lysosome fusion and/or acidification of phagosomes [12], [13], and induction of autophagy [14], [15].

There are about 18 genes encoding interferon-inducible IRG proteins in the C57BL/6 genome. The proteins have been divided into two major sequence subfamilies, informally designated GKS and GMS [16]. GMS subfamily members Irgm1, Irgm2 and Irgm3 have a most unusual methionine (GX4GMS) in the position of an otherwise universally conserved lysine (GX4GKS) in the P-loop of the G1 motif of the nucleotide-binding site. Unlike the GKS protein, Irga6, for which a crystal structure is available [17], the GMS subfamily proteins have proved difficult to purify without tags and little is known of their structural or biochemical properties. Nevertheless there is suggestive evidence from immunoprecipitated material and from fusion proteins that, despite the remarkable modification in the G1 motif, the GMS subfamily proteins are able to bind and hydrolyse GTP [18], [19].

The expression of Irgm1, together with other IRG proteins (except Irgc), is strongly induced by interferons [16]. In IFNγ-induced cells, the IRG proteins associate with cellular membranes to different extents. Irgm1 is exceptional among IRG proteins assayed in that it is exclusively membrane-associated, with no detectable cytosolic pool [20]. Although no transmembrane domain has been identified within the Irgm1 sequence, the protein behaves like an integral membrane protein in resistance to extraction with high salt, sodium carbonate or urea [20]. By immunofluorescence, native, IFNγ-induced Irgm1 localises strongly to Golgi membranes and to a lesser extent to unidentified cytoplasmic membrane systems [19], [20]. The Golgi localisation depends on a predicted amphipathic helix in the C-terminal domain of the protein, the αK-helix [20], but the basis for localisation to other cytoplasmic membrane systems has not been studied. Upon latex-bead phagocytosis, native Irgm1 rapidly re-localises in IFNγ-induced fibroblasts and macrophages to F-actin-rich plasma membrane ruffles associated with phagocytic cups, and remains associated with the phagosomes as they mature into phagolysosomes and become LAMP1 positive [20]. Uptake of M. bovis BCG by macrophages stimulates similar relocation of Irgm1 to the bacterial phagosome and phagolysosome [19].

In this study we examine in detail several aspects of the localisation of Irgm1 to cytoplasmic compartments. Building on the earlier observation [20] that the 20-residue amphipathic αK helix of Irgm1 is sufficient to target EGFP to the Golgi apparatus, we show that this sequence can also target EGFP to a cytoplasmic membrane system identified as lysosomes and late endosomes, but not to latex-bead phagosomes. We show that the αK helix could be also used in the form of a synthetic biotinylated peptide to target a labelled streptavidin complex to Golgi membranes in fixed, permeabilized cells. This biotin-peptide probe also stained cytoplasmic organelles outside the Golgi that we were able to show corresponded to late endosomes and lysosomes. This novel association proved to reflect the previously documented non-Golgi cytoplasmic localisation of native, IFNγ-induced Irgm1, which had not been located to a specific compartment [20]. Irgm1 has thus a constitutive association with the endolysosomal membrane system in addition to the Golgi apparatus, a unique property among IRG proteins tested so far. The lysosomal localisation of Irgm1 is mediated by the same amphipathic helix that mediates Golgi targeting.

We observed earlier that N-terminally EGFP-tagged, and C-terminally FLAG-tagged Irgm1 both mis-localise in cells, but the observations were not followed up and the compartments involved were not identified [20]. We now show that N and C terminal EGFP tags on Irgm1 both lead to loss of Golgi localisation and localisation to other membrane compartments. The mis-localisation of tagged Irgm1 is dependent on nucleotide binding, and we suggest that Golgi localisation is a default state probably associated with an inactive GDP-bound state of the protein. The mis-localisation of Irgm1 N- or C-terminally tagged with EGFP suggests that functional experiments employing such constructs [13]–[15], [19] may need to be interpreted cautiously.

## Results

### IFNγ-Induced Irgm1 Localises to Golgi Apparatus and Late Endocytic/Lysosomal Compartments

We previously reported that IFNγ-induced Irgm1 (LRG-47) is exclusively membrane-bound and localises in large part to the Golgi apparatus in L929 fibroblasts, TIB-75 hepatocytes and Raw 264.7 macrophages. This localisation is mediated by an amphipathic helix near the C-terminus [20] We noted that IFNγ-induced Irgm1 has a further cytoplasmic signal throughout the cell periphery of which the subcellular localisation remained to be identified. In the present study, we employed the microscopically favorable murine embryonic fibroblasts (MEFs) to characterise the intracellular localisation of Irgm1 in more detail.

**Figure 1:**
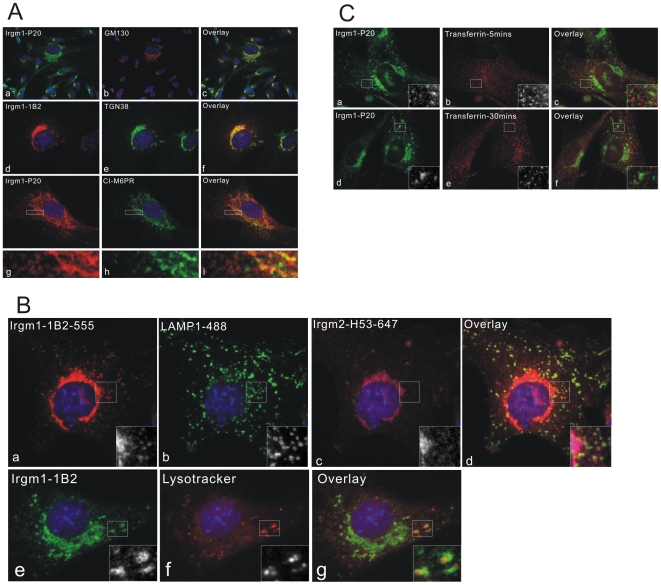
Localisation of IFNγ-induced endogenous Irgm1. (A) MEFs were treated with 200 U/ml IFNγ for 24 hours, fixed and stained for Irgm1 with goat antiserum P20 (a–c, g–l) or mouse monoclonal antibody 1B2 (d–f) (both directed against Irgm1) and the indicated marker proteins. Irgm1 accurately co-localised with GM130 (a–c) and TGN38 (d–e), and localised adjacent to, or partially co-localised with, the CI-M6PR positive compartments (g–i). (B) MEFs were treated with 200 U/ml IFNγ for 24 hours. Cells were either fixed and stained for Irgm1, LAMP1 and Irgm2 using 1B2, 1D4B, and H53 immunoreagents, respectively (a–d), or further incubated with 50 nM LysoTracker Red DND-99 for 30 minutes in complete medium, and stained for Irgm1 with 1B2 antibody (e–g). Irgm1 was found to associate with LAMP1 positive compartments (a–d) and accumulate around the LysoTracker enriched compartments (e–g) in addition to Golgi localisation. Irgm2 (c) localises exclusively to the Golgi apparatus. (C) After treatment with 200 U/ml IFNγ for 24 hours, MEFs were incubated with Alexa-Fluor-546-labelled transferrin for 5 minutes (a–c) or pulsed with labelled transferrin for 10 minutes, then chased for 30 minutes (d–f). Cells were then fixed and stained for Irgm1 with P20 goat antiserum. The granular signals from Irgm1 were not found convincingly associated with transferrin-positive compartments.

In IFNγ treated MEFs, both goat polyclonal anti-Irgm1 antiserum P20 and the mouse monoclonal anti-Irgm1 antibody 1B2 gave a focused adnuclear signal in all cells, though of variable intensity, and additional distributed granular signals, more or less conspicuous in different cells (Figure 1). High and low Irgm1 staining intensity for the two compartments were uncorrelated (Supplementary Table S1). As previously reported [20], the adnuclear signal accurately overlapped with both *cis*-Golgi matrix protein GM130 (Figure 1A panels a–c) and *trans*-Golgi/*trans*-Golgi network protein TGN38 (Figure 1A panels d–f). Less accurate co-localisation was observed with cation-independent mannose 6 phosphate receptor (CI-M6PR), which is predominantly localised in late endosomes (Figure 1A panels g–i); Irgm1 localises rather to places adjacent to or partially overlapping with CI-M6PR positive compartments. Furthermore, the more widely distributed punctate signal of Irgm1 accurately co-localised with the late endosome/lysosome marker LAMP1 (Figure 1B panels a–d)). By comparison, Irgm2 localised exclusively to Golgi apparatus, as reported earlier [1] with no extra signal overlapping with LAMP1 (Figure 1B panel c). To confirm the apparent lysosomal localisation of Irgm1, the acidotropic dye LysoTracker was loaded and cells were co-stained with anti-Irgm1 antibody 1B2. Irgm1 was found to accumulate around the LysoTracker enriched compartments (Figure 1B panel e–g). By comparison, endogenous Irgm1 rarely co-localised with transferrin-labelled early and recycling endocytic compartments (Figure 1C). Taken together, we conclude that native, IFNγ-induced Irgm1 is variably associated with acidified late endocytic/lysosomal compartments in addition to Golgi apparatus.

**Figure 2:**
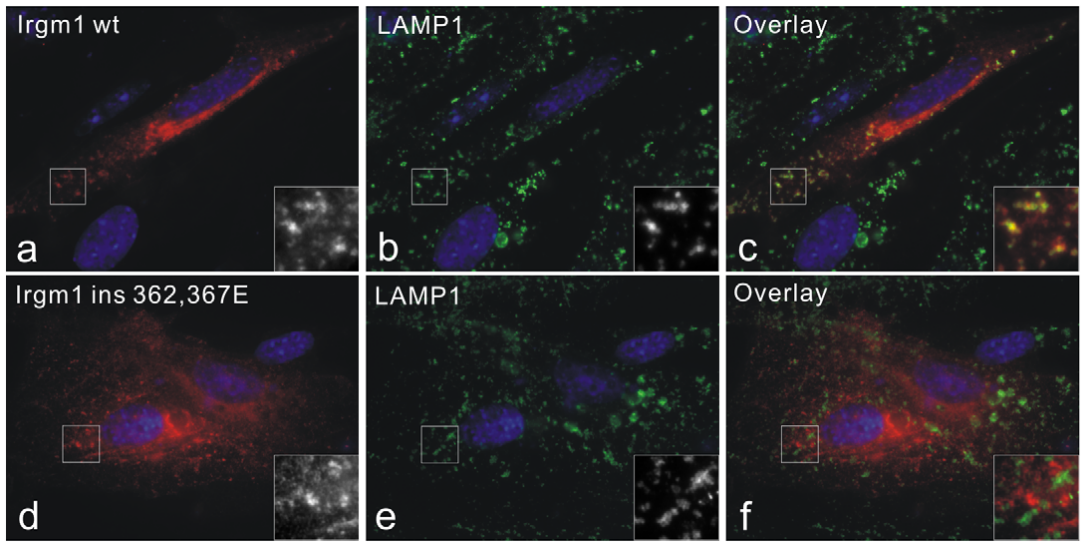
Amphipathic αK helix is responsible for lysosomal targeting of full length Irgm1 protein. MEFs were transfected with plasmids encoding either Irgm1 wild type (a–c) or the ins 362, 367E mutant (d–f) for 24 hours in the absence of IFNγ. Cells were then fixed and stained for Irgm1 and LAMP1. Wild type Irgm1 is strongly associated with the LAMP1 positive compartment, while Irgm1 ins 362, 367E mutant showed granular signals throughout cytoplasm which do not overlap with LAMP1 signals. Nuclei were labelled with DAPI.

We previously reported that a predicted amphipathic helix near the C terminus of Irgm1 (named αK helix after the equivalent element in the known Irga6 structure) is responsible for the localisation of the protein to Golgi membranes [20]. The αK helix is a true targeting motif since, as a C-terminal tag, it was sufficient to target EGFP accurately to the Golgi apparatus as defined by GM130 staining [20]. Disruption of amphipathicity by glutamate insertion into the helical region abolished the Golgi localisation in both EGFP-Irgm1 αK construct and Irgm1 full-length protein. Since we now show that IFNγ-induced Irgm1 also localises to lysosomes, we ask whether the native αK helix of full-length Irgm1 is also necessary for lysosomal localisation. Full length Irgm1 and the ins 362,367E Irgm1 mutant, in which the amphipathicity of the αK targeting helix was destroyed by two glutamate insertions [20], were transiently expressed in un-induced MEFs (Figure 2). Wild type Irgm1 showed both Golgi and lysosomal association like the endogenous IFNγ-induced protein (Figure 2 panels a–c). In these cells the co-localisation with LAMP1 was even more striking than in IFNγ-induced cells. Irgm1 ins 362, 367E mutant was distributed as dotty structures throughout the cytoplasm that showed neither Golgi nor lysosomal localisation (Figure 2 panels d–f and [20]). This new localization, which was not identified, may indicate the present of another membrane-targeting motif in Irgm1 and deserves further analysis. Thus the native amphipathic αK helix is required for both Golgi and lysosomal targeting of full length Irgm1 protein.

### The Amphipathic αK Helix Near the C-Terminus of Irgm1 Shows Golgi and Lysosomal Targeting Property

**Figure 3:**
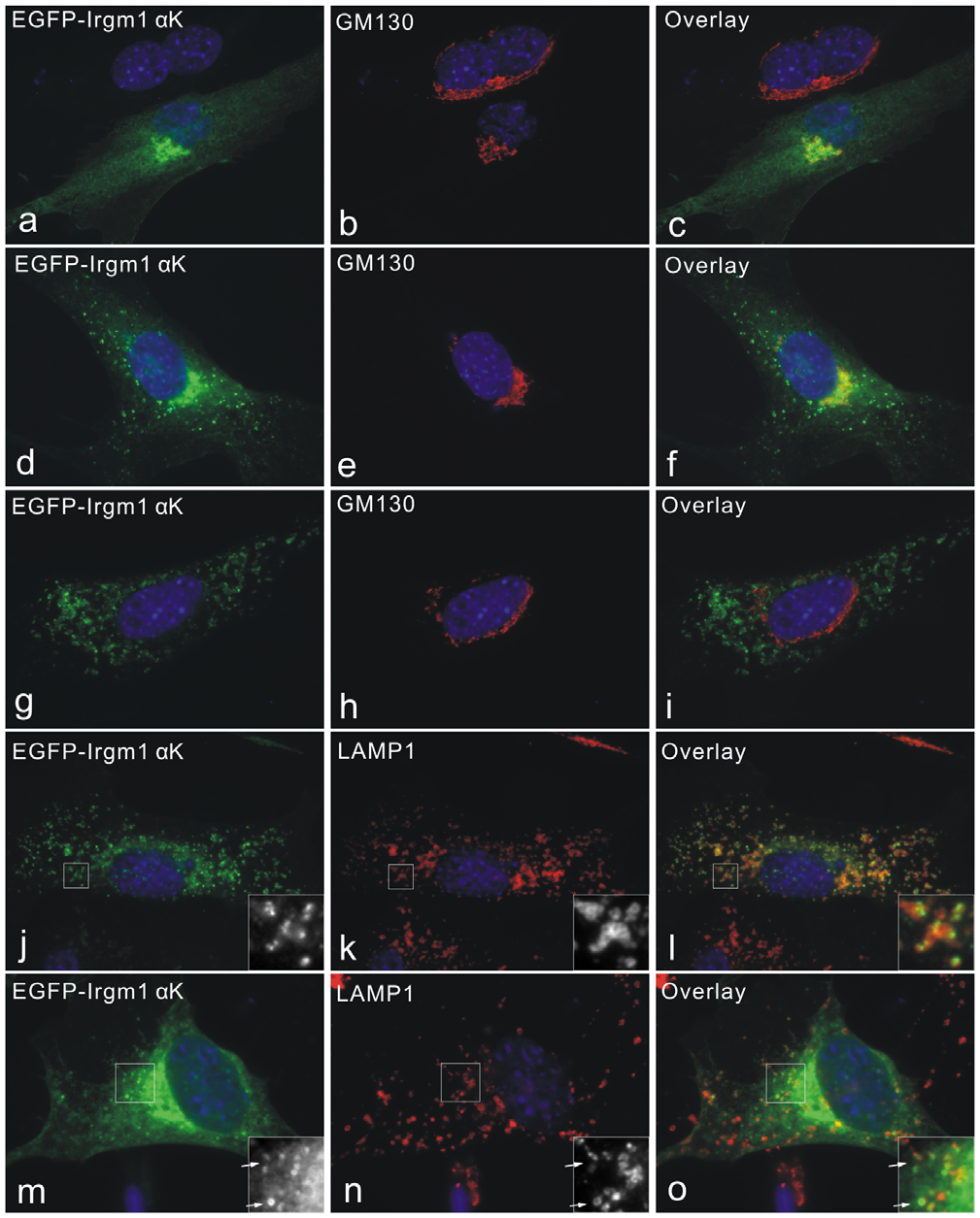
EGFP-Irgm1 αK localises to both Golgi and lysosomes. MEFs were transfected with an expression plasmid encoding EGFP-Irgm1 αK in the absence of IFNγ (20). 24 hours later cells were fixed and stained for either GM130 (a–i) or LAMP1 (j–o). Transfected cells stained with GM130 showed 3 rather different staining patterns for EGFP-Irgm1 αK: clear Golgi staining with only diffuse staining elsewhere (a–c), clear Golgi staining and punctate staining elsewhere (d–f), and only punctate staining without significant Golgi staining (g–i). When stained for LAMP1, cytoplasmic dots of EGFP-Irgm1 αK, without (j–l) or with (m–o) Golgi localisation were more or less strongly associated with LAMP1-positive vesicles. Nuclei were labelled with DAPI.

We previously observed that the EGFP-Irgm1 αK construct was often associated with a cytoplasmic compartment in addition to Golgi-marker-positive compartments in some transfected cells [20]. We therefore now analysed the intracellular localisation of the EGFP-Irgm1 αK construct in more detail in MEFs (Figure 3). We observed that when this construct was highly expressed in cells the construct accumulated in aggregates in the cytoplasm, sometimes associated with obviously cytopathic changes such as cell shrinkage and rounding up (not shown). The following observations therefore apply only to cells showing relatively low expression levels of the construct and no evident cytopathic changes. In such cells there was considerable heterogeneity in the expression pattern of the EGFP-Irgm1 αK construct. Figure 3 shows the differential localization of the EGFP-Irgm1 αK construct in individual cells from a single transfected population. When co-stained for GM130 as a Golgi marker, in some transfected cells the EGFP-Irgm1 αK construct localised largely to the Golgi apparatus with only a weak, distributed, diffuse background over the rest of the cytoplasm (Figure 3 panels a–c). In other cells EGFP-Irgm1 αK localised to the Golgi apparatus as defined by GM130 co-localisation but additionally showed a more or less punctate distribution in the cytoplasm (Figure 3 panels d–f). In further transfected cells there was no co-localisation of transfected EGFP-Irgm1 αK with GM130 at all and all the transfected construct was present in cytoplasmic punctae (Figure 3 panels g–i). Some but not all of the cytoplasmic punctae co-localised with the lysosomal protein LAMP1 (Figure 3 panels j–o). Thus the αK amphipathic helix can target EGFP *in vivo* to both Golgi and lysosomal compartments. Additional cytoplasmic accumulations of EGFP-Irgm1 αΚ remain unidentified. The phenotypic diversity between cells is unexplained, but seems also to reflect in part the behaviour of native induced Irgm1 shown in Figure 1.

**Figure 4:**
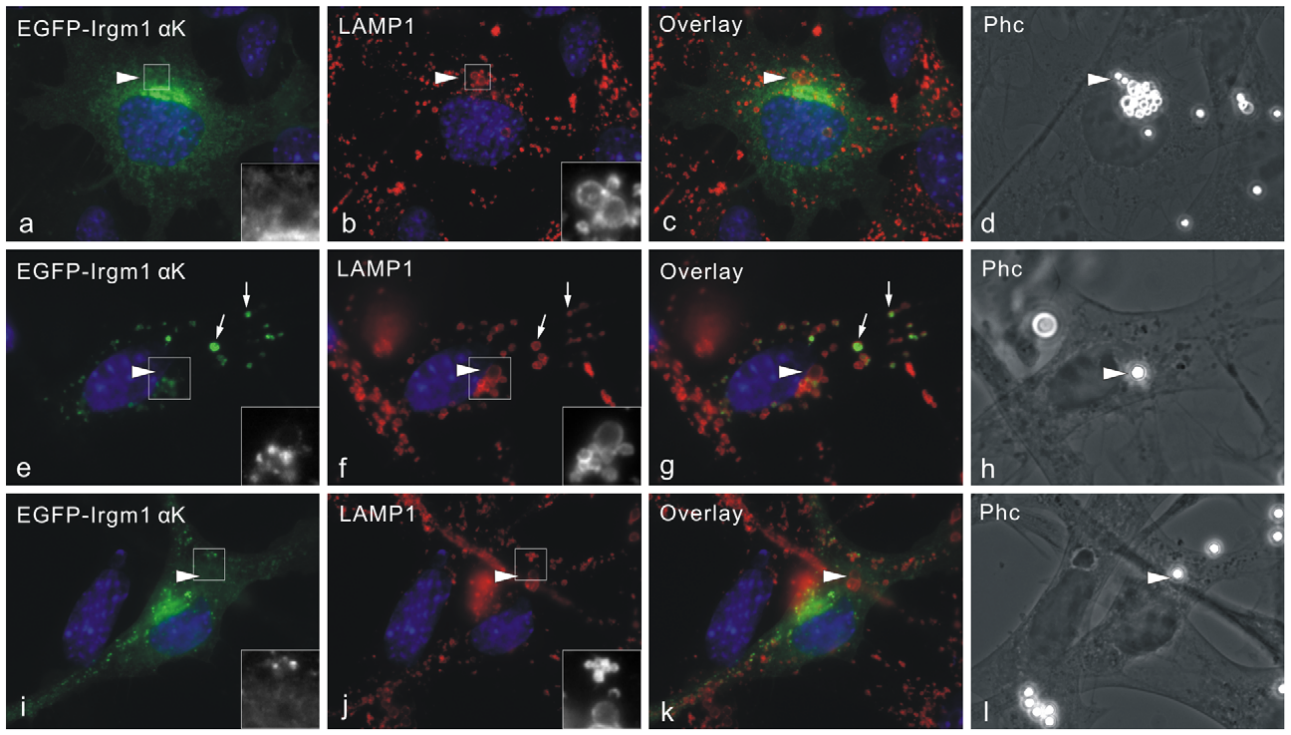
EGFP-Irgm1 αK is not recruited to phagosomes. EGFP-Irgm1 αK was transiently transfected into MEFs for 24 hours in the absence of IFNγ. During transfection, cells were incubated with 2-µm latex beads overnight. Cells were then fixed and stained for LAMP1. EGFP-Irgm1 αK was not associated with latex bead phagosomes (arrowheads), whether the association with other cellular compartments was Golgi only (a–d), lysosomal only (e–h) or both Golgi and lysosomal (i–l). Despite not associating with latex bead phagosomes, EGFP-Irgm1 αK associated normally with the LAMP1 compartment elsewhere (arrows). Nuclei were labelled with DAPI.

Since native Irgm1 is rapidly recruited to latex-bead phagosomes ([20] and see below), we next asked whether the αK helix also showed phagosomal accumulation. EGFP-Irgm1 αK was expressed by transfection in MEFs and cells were incubated with 2-µm latex beads to induce phagocytosis. Even though co-localisation was seen between EGFP-Irgm1 αK and LAMP1 in cells taking up beads (Figure 4 panels e, f, g arrows), latex bead phagosomes remained negative or very weakly stained for EGFP-Irgm1 αK (Figure 4 panels a, e, i). Thus the Irgm1 αK helix alone is not sufficient for the active accumulation of full-length Irgm1 on the phagosome.

### The Overall Amphipathicity of the αK Helix and the Identity of the Non-Hydrophobic Residues Both Contribute to the Golgi and Lysosome Localisation of the αK Helix

**Figure 5:**
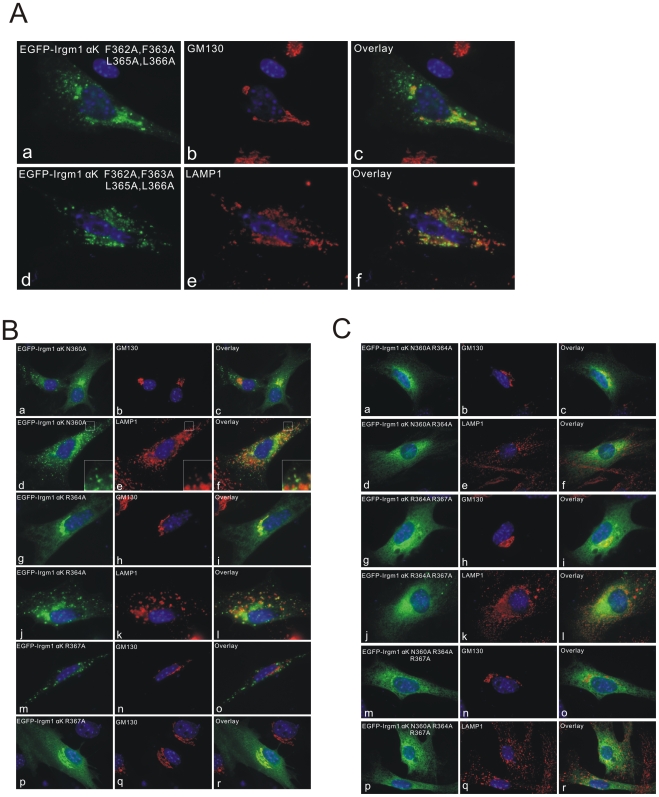
Alanine mutagenesis scan of EGFP-Irgm1 αK. EGFP-Irgm1 αK quadruple mutant (F362A F363A L365A L366A; Figure 6A), single mutants of the hydrophilic residues (N360A;R364A;R367A; Figure 6B including enlargements (for d, e, f)), or combined mutants of more than one hydrophilic residues (N360A, R364A; R364A, R367A; N360A, R364A, R367A; Figure 6C) were transfected into MEFs for 24 hours. Cells were then fixed and staining for GM130 or LAMP1. Only when more than one hydrophilic residue was mutated to alanine, the EGFP-Irgm1 αK no longer localised to Golgi or lysosomes. These mutants localised to unidentified endomembrane-like structures or are delocalized and possibly cytosolic. Nuclei were labelled with DAPI.

To investigate the possible mechanisms of subcellular localisation of Irgm1, an alanine mutagenesis scan was performed based on the targeting construct EGFP-Irgm1 αK. Single mutations of hydrophobic residues (C356A; I358A; V359A; F362A; F363A; L365A; L366A not shown) double mutations (F362A F363A and L365A L366A not shown) and even the quadruple mutant (F362A F363A L365A L366A, Figure 5A) had no marked influence on EGFP-Irgm1 αK localisation. Cells with predominantly Golgi-localised (not shown) mixed Golgi and LAMP1 (Figure 5A panel a–c) or predominantly LAMP1-associated (Figure 5A panels d–f) EGFP-αK mutant signals, were seen, as with the wild-type αK (Figure 4). Single mutations of the hydrophilic residues also had no effect on localisation. Again, cells with predominantly Golgi or predominantly LAMP1 localisation were seen (N360A Figure 5B, panels a–c, d–f;R364A Figure 5B, panels g–i, j–l);R367A Figure 5B, panels m–o, p–r). However, if more than one charged or polar residue was mutated to alanine both Golgi and lysosomal localisation were completely abolished (N360A, R364A; R364A, R367A; N360A, R364A, R367A; Figure 5C). These latter mutants were distributed in an unspecific manner throughout the cytoplasm and were possibly largely cytosolic. The increasing intensity generally around the Golgi area, though not concentrated on Golgi membranes, probably simply reflects the extra thickness of the cytoplasm at this site. These results suggest that both the overall amphipathicity of the αK helix and the identity of the non-hydrophobic residues contribute together to the membrane and Golgi/lysosome localisation of the αK helix, and it is possible that the specificity for Golgi and lysosomal membranes is determined by the identity of the polar residues.

### Synthetic αK Amphipathic Peptide Mimics the Localisation of Endogenous Irgm1

**Figure 6:**
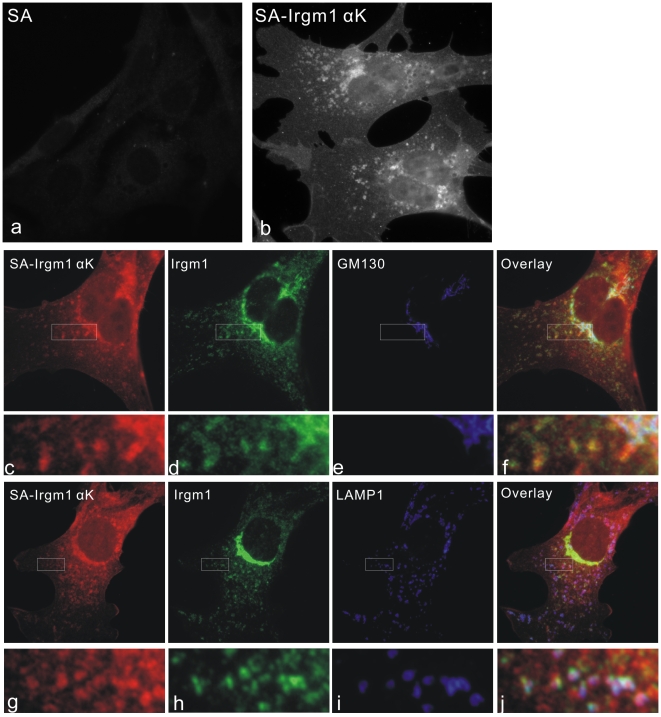
Synthetic αK amphipathic peptide mimics the localisation of endogenous Irgm1. MEFs were treated with 200 U/ml IFNγ for 24 hours and then fixed with 3% PFA in PBS. Cy3-streptavidin-peptide solutions (0.33 µM streptavidin) were prepared as described in Materials and Methods. Antibodies against the indicated proteins were added to the streptavidin-peptide solution and the mixtures used as primary reagents in immunofluorescence staining. Streptavidin (0.33 µM final) alone (a) was used as control. Image (a) and (b) were taken at the same exposure time. Colocalisation was seen between streptavidin-Irgm1 αK peptide and endogenous Irgm1 at both Golgi apparatus and lysosomes.

Biochemical studies showed that Irgm1 behaves like an integral membrane protein, even though no transmembrane domain has been identified within the protein sequence [20]. In the same study, it was also demonstrated that Irgm1 exclusively associates with the membrane fraction of the cell [20]. Attempts to purify untagged recombinant Irgm1 protein failed due to insolubility (not shown). To investigate the possible mechanisms of Irgm1 localisation, a peptide corresponding to the targeting amphipathic sequences (SK*LRLMTCAIVNAFFRLLRFLPCVCC) of Irgm1 was synthesized. The lysine in position 2 (K*) was covalently conjugated with biotin. Thus the peptide could be loaded onto fluorochrome-conjugated streptavidin protein, which has four biotin binding sites, to form a tetrameric, fluorescent peptide-streptavidin complex. The localisation of this complex was then examined directly on fixed, permeabilized cells. The procedures for making and using this tetramer system are described in Materials and Methods. Figure 6 shows the staining pattern of Cy3 labelled streptavidin-Irgm1 αK tetrameric complexes in MEFs. Cy3-labeled streptavidin alone was used as control and pictures were taken with the same exposure time (Figure 6 panels a, b). The streptavidin-Irgm1 αK complex staining shows both perinuclear and vesicular structures corresponding to the localisation of endogenous Irgm1 protein (Figure 6 panels c, d, g, h). GM130 (Figure 6 panel e) and LAMP1 (Figure 6 panel i) were additionally used to identify the Golgi apparatus and lysosomes. Streptavidin-Irgm1 αK complexes showed striking co-localisation with full length Irgm1 at both Golgi apparatus and lysosomes. Co-localisation with GM130 was seen also in cells not induced with IFNγ (data not shown), suggesting that the targeting of the complex was due to a component unresponsive to IFNγ and not due to direct association with endogenous Irgm1. There was, however, more diffused staining with the synthetic complexes, perhaps due to the intrinsic hydrophobic character of the peptide. These results demonstrate that the streptavidin-αK peptide complex closely resemble the localisation of endogenous Irgm1 and provides a valuable novel method to investigate the function of targeting motifs of proteins.

### Both N and C Terminal EGFP-Tags Cause Mislocalisation of Irgm1

**Figure 7:**
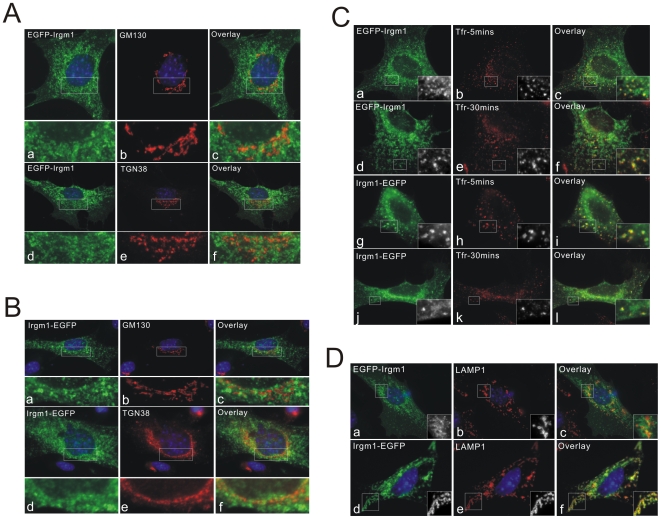
Localisation of EGFP-tagged Irgm1. (A) N-terminally EGFP-tagged Irgm1 (EGFP-Irgm1) or (B) C-terminally EGFP-tagged Irgm1 (Irgm1-EGFP, pF25) were transfected into MEFs. 24 hours later, cells were fixed and stained for GM130 (a–c) and TGN38 (d–f). Both constructs were absent from the Golgi apparatus. Nuclei were labelled with DAPI. (C) EGFP-Irgm1 (a–f) or Irgm1-EGFP (pF25, g–l) were transfected into MEFs. 24 hours later cells were incubated with Alexa-Fluor-546-labelled transferrin for 5 minutes (a–c, g–i) or pulsed with labelled transferrin for 10 minutes, then chased for 30 minutes (d–f, j–l). Cells were then fixed in 3% PFA in PBS. The vesicular and dotty signals from EGFP-Irgm1 and Irgm1-EGFP strongly overlap with transferrin-labelled early and recycling endosomes. (D) EGFP-Irgm1 (a–c) or Irgm1-EGFP (d–f, pF25) were transfected into MEFs. 24 hours later, cells were fixed and stained for LAMP1. Irgm1-EGFP was strongly colocalised with LAMP1 while lysosomal association of EGFP-Irgm1 was largely but not completely abolished. Nuclei were labelled with DAPI.

The proposed autophagy-induction function of Irgm1 is largely based on over-expression experiments using C-terminally EGFP-tagged Irgm1 [14], [15]. However, we observed earlier that both N and C terminally tagged Irgm1 misbehave in cells [20]. To investigate to which compartments tagged Irgm1 localises in cells, both N and C terminally EGFP-tagged Irgm1 were expressed in MEFs and co-stained with Golgi markers, endocytosed transferrin and LAMP1 (Figure 7). EGFP-Irgm1 and Irgm1-EGFP (pF25, [13]–[15]) both showed diffuse vesicular and dotty expression patterns throughout the cytoplasm. Co-localisation with the Golgi protein GM130 and TGN38 was abolished in all cells (Figure 7A and B). Many of these vesicular and dotty signals strongly co localised with transferrin-labelled early endosomes and recycling endosomes (Figure 7C). As described earlier, no co-localisation was found between native IFNγ-induced Irgm1 and early and recycling endosomes (Figure 1C). The Irgm1-EGFP construct showed strong co-localisation with LAMP1 while for EGFP-Irgm1 the lysosomal association was generally reduced (Figure 7D). Since Irgm1 associates with membranes independently of IFNγ-induced factors [20], we asked whether IFNγ could influence the mislocalisation of EGFP-Irgm1 and Irgm1-EGFP. The two constructs were transfected into MEFs simultaneously induced with IFNγ. Both EGFP-Irgm1 and Irgm1-EGFP mislocalised also in IFNγ-induced cells (data not shown). In conclusion, neither N nor C terminally EGFP-tagged Irgm1 localises to Golgi membranes, while both continue to localise to LAMP1 positive compartments and mislocalise to differing extents to early and recycling endocytic compartments.

C-terminally Irgm1-EGFP-tagged constructs have been used elsewhere both for studies of autophagy [14], [15] and mycobacterial resistance [12], [19] in IFNγ-induced RAW264.7 macrophages. These cells are not ideal for immunofluorescence localization because of their complex 3-dimensional morphology. Nevertheless we have been unable to confirm the claimed Golgi localization of C-terminally EGFP-tagged Irgm1 in these cells (Supplementary Figure S1 panels a–l). The construct is localized throughout the cytoplasm and is hard to resolve away from the Golgi. However the Golgi itself is very well defined by GM130 and there is no tendency towards concentration or enrichment of the Irgm1-EGFP corresponding to the Golgi, either in resting or IFNγ-induced RAW264.7 cells. In contrast, the localization of native, IFNγ-induced Irgm1 in RAW cells, is closely associated with Golgi membranes, as it is in MEFs (Figure S1, panels m–p). Thus the mislocalisation of N-terminally EGFP-tagged Irgm1 is not a cell-type-specific characteristic of MEFs, but a fixed characteristic of the construct.

### Mislocalisation of Irgm1 by EGFP Tagging Is Nucleotide Dependent

**Figure 8:**
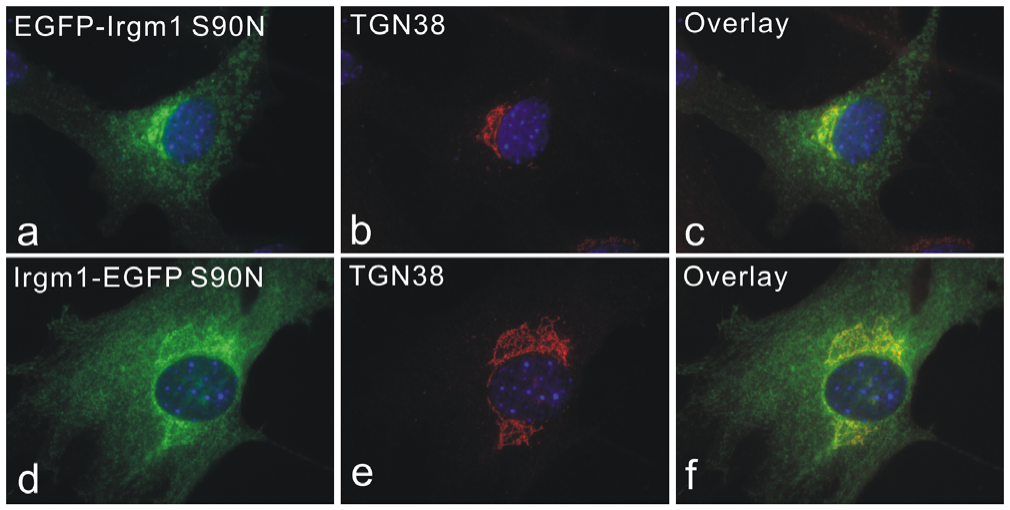
Mislocalisation of EGFP-tagged Irgm1 is nucleotide-dependent. EGFP-Irgm1 S90N (a–c) and Irgm1-EGFP S90N (d–f) were transfected into MEFs for 24 hours. Cells were then fixed and stained for TGN38. Both constructs co-localised with TGN38, although a significant proportion of transfected proteins displayed additional cytoplasmic signals. Nuclei were labelled with DAPI.

Irgm1 is associated with Golgi membranes independently of nucleotide binding [20]. To test whether nucleotide binding influences the mislocalisation of Irgm1 caused by EGFP tagging, the Irgm1 nucleotide binding deficient mutant (S90N) was tagged with EGFP at N or C terminus and expressed in MEFs. Both fusion proteins accurately co-localised with Golgi protein TGN38 and GM130 (Figure 8 and not shown) but more cytoplasmic reticular and vesicular signals were also seen. The expression patterns of EGFP-Irgm1 (S90N) and Irgm1-EGFP (S90N) were similar to the localisation of untagged nucleotide-binding deficient Irgm1 (S90N) [20].

### Phagosomal Accumulation of Irgm1 Is Nucleotide Dependent, but IFNγ Independent

**Figure 9:**
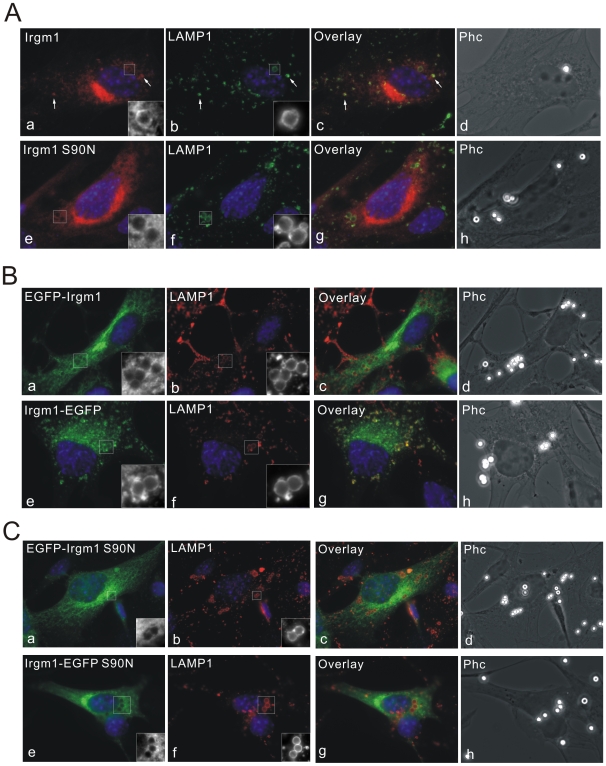
Phagosomal accumulation of Irgm1 is nucleotide-dependent, but IFNγ-independent. (A) Irgm1 wild type (a–d) and S90N mutant (e–h) were transfected into MEFs in the absence of IFNγ for 24 hours. During transfection, cells were incubated with 2-µm latex beads overnight. Cells were then fixed and stained for Irgm1 and LAMP1. Irgm1 accumulated around the latex beads phagosomes (seen as refringent spheres) while the loss of the nucleotide-binding site completely abolished the phagosomal accumulation. Arrows indicate the co-localisation of transfected wild type Irgm1 with LAMP1 positive compartments outside latex-bead phagosomes. (B) EGFP-Irgm1 or Irgm1-EGFP were transfected into MEFs for 24 hours in the absence of IFN**γ**. During transfection, cells were also incubated with 2-µm latex beads overnight. Cells were then fixed and stained for LAMP1. Irgm1-EGFP accumulated around the latex bead phagosomes while EGFP-Irgm1 did not. (C) The same experiments were performed as in (B) but using the nucleotide-binding deficient mutants (S90N) of N- and C-terminally EGFP-tagged Irgm1. Both mutants were absent from latex bead phagosomes. Nuclei were labelled with DAPI.

In L929 fibroblasts and Raw264.7 macrophages, IFNγ-induced Irgm1 is rapidly recruited to active plasma membrane upon phagocytosis and remains associated with phagosomes as they mature [20]. It was also reported that Irgm1 associates with Golgi apparatus in an IFNγ and nucleotide independent manner [20]. We therefore asked whether Irgm1 can accumulate on latex bead phagosomes independently of IFNγ and if so, whether the association with phagosomes is regulated by nucleotide binding. Irgm1 wild type and nucleotide binding deficient mutant Irgm1(S90N) were expressed in MEFs in the absence of IFNγ, and phagocytosis was initiated by incubating with 2-µm latex beads overnight. Wild type Irgm1 strongly associated with latex bead-phagosomes while accumulation of Irgm1(S90N) on the phagosomal membrane was much reduced (Figure 9A). Transfected Irgm1 also co-localised with LAMP1 positive compartments in unstimulated cells taking up latex beads (Figure 9A arrows), as well as in cells not involved in phagocytosis (not shown). Irgm1(S90N) still showed Golgi localisation but lysosomal distribution was largely abolished (Figure 9A panels e–h). Therefore, the accumulation of Irgm1 on phagosomes is nucleotide dependent, but IFNγ independent.

As shown above, Irgm1-EGFP showed strong lysosomal association, while EGFP-Irgm1 did not. Consistent with this observation, Irgm1-EGFP accumulated strongly on latex bead phagosomes whereas EGFP-Irgm1 was absent or only very weakly present on phagosomes (Figure 9B). Like untagged Irgm1, the accumulation of Irgm1-EGFP on phagosomes was also regulated by nucleotide binding. Irgm1(S90N)-EGFP no longer associated with latex bead phagosomes (Figure 9C).

## Discussion

The different members of the mouse IRG resistance protein family show different associations with cellular membranes. Unlike all the other IRGs, which have a more or less prominent cytosolic component, Irgm1 has been found exclusively in membrane-bound form in cells. Although no transmembrane domain could be identified in the sequence, Irgm1 behaves like a transmembrane protein in biochemical assays [20]. In L929 fibroblasts, TIB-75 hepatocytes and Raw 264.7 macrophages, Irgm1 localises conspicuously to *cis-* and *medial-*Golgi membranes with an additional cytoplasmic signal [20]. In the present study, by using TGN38 as a *trans-*Golgi and *trans-*Golgi network (TGN) marker, the Golgi localisation domain of Irgm1 is extended to these compartments (Figure 1A). We also show that the additional cytoplasmic signals of Irgm1 overlap with those of LAMP1, which marks late endosomes and lysosomes. This observation is confirmed by co-localisation of Irgm1 with compartments enriched in the acidotropic dye LysoTracker (Figure 1B). We noted that, compared to the universal Golgi localisation of Irgm1, the signal intensity of Irgm1 in the LAMP1-positive compartments varied in different individual cells, from conspicuous to hardly detectable. Since MEFs are isolated from mouse embryos and constitute a developmentally and morphologically heterogeneous population, the variable degrees of lysosomal association of Irgm1 could correlate with different states of individual cells, with stages of the cell cycle or with other undefined factors. Native endogenous Irgm1 is scarcely if at all detectable on early and recycling endosomes defined by early and late uptake of fluorochrome-labelled transferrin (Figure 1C). Taken together, we show here for the first time that the IFNγ-induced endogenous Irgm1 localises to the late endocytic/lysosomal (LAMP1-positive) compartments in addition to the Golgi apparatus in the absence of infection. Irgm1 is the first IRG protein to be shown to localise to the endosomal/lysosomal membrane system.

We further showed in MEFs that both N and C terminally EGFP-tagged Irgm1 (EGFP-Irgm1 and Irgm1-EGFP, respectively) localise differently from native Irgm1. Neither form of tagged protein localized to the Golgi apparatus (Figure 7A and 7B). In a further difference from the native protein, Irgm1-EGFP showed significant association with LAMP1-positive compartments in all transfected cells, in contrast to the variable lysosomal association of IFNγ-induced endogenous Irgm1 (Figure 7D). Unlike endogenous Irgm1, however, Irgm1-EGFP also localized to early and recycling endosomes defined by the uptake of transferrin (Figure 7C). N-terminally tagged Irgm1 showed still more divergent behaviour. In addition to failing to localise to the Golgi, EGFP-Irgm1 also associated with LAMP1-positive compartments very weakly, if at all. The construct was distributed mainly in early and recycling endosomes as well as some other undefined, non-Golgi, non-LAMP1-positive, vesicular structures (Figure 7C and 7D). The observations on EGFP-Irgm1 have been confirmed in Raw264.7 macrophages (Supplementary Figure S1). It has been reported that Irgm1-EGFP (pF25) co-localised with MDC and LC3 positive compartments, suggesting that Irgm1 associates with autophagosomes and therefore has a pro-autophagy function in macrophages [14], [15]. The present results suggest that the association of EGFP-tagged Irgm1 with autophagosomes may result from transfer of the protein from lysosomes or other compartments of the endocytic system, but not from the Golgi apparatus, where the fusion protein fails to localize. Because of the altered localization of the EGFP-Irgm1 constructs, the association of endogenous Irgm1 with autophagic compartments will bear careful examination.

We found that both N and C terminally EGFP-tagged nucleotide-binding deficient Irgm1(S90N) localised to the Golgi with little if any mislocalisation (Figure 8), indicating that the targeting of Irgm1 to different compartments is controlled by the nucleotide binding state of the protein. Golgi localisation presumably reflects the inactive or GDP-bound state of the protein since nucleotide-binding deficient Irgm1 (S90N), whether tagged with EGFP or not, localises to Golgi. Correspondingly, the endosomal and lysosomal localisation of EGFP-tagged Irgm1 may reflect the activated or GTP-bound form of Irgm1, suggesting that the tags may cause constitutive or inappropriate activation, or inhibit nucleotide exchange. We have recently shown that another IRG protein, Irga6, normally rests in cytoplasmic compartments in the inactive state, but depends on continuous regulatory interactions with Irgm1, Irgm2 and Irgm3 to inhibit spontaneous activation [8]. IRG proteins belong to the dynamin-like large GTPase family, members of which show common properties of GTP-dependent oligomerisation and cooperative activity [21]. We have shown that Irgm1 can interact with itself in a yeast 2-hybrid system, and self-interaction is abolished by destroying the nucleotide-binding motif (S90N) [8]. It may be that EGFP-tagged Irgm1 proteins form dimers in cells [22] resulting in premature activation through the enforced proximity of two G-domains.

We showed recently that Irgm1, as well as its close relatives, Irgm2 and Irgm3, function primarily to regulate the activation of other “effector” members of the IRG protein family such as Irga6, Irgb6 and Irgd [8]. There we argued that the regulatory interactions should occur primarily at cytoplasmic membranes rather than free in the cytosol. An obvious problem with that argument, however, was the apparent absence of a regulatory IRGM protein on the endo-lysosomal system. With the data of the present report, however, endolysosomal membranes can now potentially be included in regulatory activity mediated by Irgm1.

Golgi localisation of Irgm1 is mediated by the predicted amphipathic αK helix near the C-terminus, a sequence that can also target EGFP to the Golgi [20]. We show here that the same sequence will also target EGFP to lysosomes defined by LAMP1. IFNγ-induced endogenous Irgm1 also showed different levels of lysosomal association, with strong lysosomal signals in some cells and hardly detectable signals in other cells. The signal intensities in the two compartments varied independently of each other (Supplementary Table S1). The mechanism for this ambiguous, possibly dynamic localisation behaviour is unclear: as noted above, it may correlate with spontaneous activation of the GTPase cycle.

The Irgm1 αK helix was required not only for targeting EGFP to Golgi and lysosomes, but also for the correct localisation of full-length protein ([20] and this report). A mutant with glutamate insertions to disrupt the amphipathicity of αK helix in full-length Irgm1 distributed as uncharacteristic dotty structures throughout the cytoplasm showing no overlap with GM130 or LAMP1 ([20] and Figure 2). The implication of this observation is that Golgi and lysosomal localisation of full-length Irgm1 depends on the amphipathicity of the αK helix. However, although Irgm1 αK helix is sufficient to target EGFP protein to Golgi and lysosomes, it is not sufficient to target EGFP to the latex bead phagosomes (Figure 4). Since accumulation of full-length Irgm1 on phagosomes depended on the integrity of the nucleotide-binding motif, it seems that another GTP-dependent conformationally active region of full-length Irgm1 must contribute to the phagosomal accumulation of the protein.

An alanine scan was performed to investigate the nature of the interaction of the EGFP-Irgm1 αK construct with Golgi or lysosomal membranes (Figure 5). Single mutations for each individual residue in the αK helical region and double/quadruple mutations for hydrophobic residues did not change the Golgi/lysosomal localisation of EGFP-Irgm1 αK. The localisation was also unaffected by single changes to charged or polar residues in the helical region. However, when two or more non-hydrophobic residues were substituted by alanines, EGFP-Irgm1 αK was distributed generally throughout the cytoplasm, overlapping neither with GM130 nor with LAMP1, and was possibly in the soluble compartment. These results suggest that both the overall amphipathicity of the αK helix and the identity of the non-hydrophobic residues contribute together to the membrane and Golgi/lysosome localisation of the αK helix. The insensitivity of the targeting specificity to exchanges of the hydrophobic residues to alanine suggests that the main function of these residues is to present a hydrophobic face and associate with the lipid component of the membrane [20]. The contribution of the non-hydrophobic residues needs further analysis as it is unclear whether the effect observed from the double/triple non-hydrophobic-residue to alanine mutations is primarily due to an increase in overall hydrophobicity or to the loss of the specific polar side-chains. In addition, there are two basic residues (K349, R351) and three large hydrophobic residues (L350, L352, M353) preceding the predicted amphipathic helix that were not analyzed in the present study. Altogether there is a high frequency of basic residues in the αK targeting sequence, the positive charges associated with the sequence may be necessary to increase the overall affinity of the sequence for the negatively charged head groups on the cytosolic face of the membrane. It is certainly not excluded that the αK targeting sequence may be targeted to bind to specific phosphoinositides as suggested recently [19], [23].

Not only Irgm1, but all mouse IRGM proteins have a membrane-targeting signal in the position of the αK helix in the Irga6 crystal structure. The αK helix from Irgm2 targets EGFP to the Golgi apparatus and the αK helix from Irgm3 targets EGFP to a reticular endomembrane system, both localisations that largely correspond to those of the respective full-length proteins [1]. Efforts to purify untagged IRGM proteins for biochemical analysis have not been successful so far. We therefore took advantage of synthetic small targeting peptides to study the subcellular localisation of IRGM proteins. The αK peptide from Irgm1 was a pioneer for this approach. There are several advantages to the peptide-streptavidin tetramer system described here. First, the targeting αK helix peptide from Irgm1 was insoluble in neutral pH buffers, but when loaded onto streptavidin protein, the solubility of the complex at neutral pH was adequate for experiment. Secondly, streptavidin is a natural tetrameric protein and each molecule has the capacity to bind four biotinylated peptides, resulting in enhanced avidity of the targeting complex to its target. Thirdly, streptavidin can be covalently labelled with fluorochromes and used directly in fluoro-cytochemistry staining. Irgm1 αK peptide has been shown in the present study to successfully mimic the localisation of endogenous full-length Irgm1 in both Golgi apparatus and lysosomal compartments in MEFs (Figure 6) and L929 cells (not shown).

While the present paper was under review a report appeared concluding that the αK targeting sequence of Irgm1 is specific for the two phosphoinositides PI(3,4)2P and PI(3,4,5)3P formed at the mycobacterial phagosome during pathogen uptake [19]. The methods and conclusions of that paper touch those of the present paper at several points and deserve comment. Firstly, that study makes extensive use of Irgm1 constructs tagged either at the N- or C-terminus with EGFP or its derivatives, but mislocalisation of such constructs was not observed. While Golgi localisation of the endogenous protein and of an EGFP-Irgm1 αK construct apparently identical to our own [20] was reported, no localisation of either to a LAMP1 compartment was reported in uninfected cells. Finally, localisation of the EGFP-Irgm1 αK construct to the mycobacterial phagosome was reported while we found no localisation of the EGFP-Irgm1 αK construct to latex bead phagosomes. One possible reconciliation of these two disparate sets of findings is the different cell types used. Tiwari and colleagues [19] employed a variety of cell types, including primary bone-marrow-derived macrophages and RAW264.7 macrophages, while our studies were largely on mouse primary embryonic fibroblasts. Our use of MEFs was dictated by the far superior properties of these cells for organelle identification and signal co-localisation. We have, however, in our own experiments seen no discrepancies so far between RAW264.7 cells and the localisations that we illustrate here with MEFs. In particular, endogenous IFNγ-induced Irgm1 clearly localised to the Golgi in RAW264.7 macrophages, while equally clearly, Irgm1-EGFP and EGFP-Irgm1 constructs did not localise detectably to the Golgi in these cells (Supplementary Figure S1).

In summary, this report documents two critical properties of Irgm1 in mouse fibroblasts, both of which should be taken seriously when working on this interesting resistance protein. Firstly, native, IFNγ-induced Irgm1 localises to endolysosomal membranes as well as to Golgi, and secondly, both N- and C-terminally EGFP-tagged Irgm1 localise abnormally and should probably not be used for functional studies on the role of Irgm1 in cells.

## Materials and Methods

### Expression Constructs

EGFP-Irgm1 αK, EGFP-Irgm1, Irgm1-EGFP (pF25), pGW1H-Irgm1, pGW1H-Irgm1 S90N, pGW1H-Irgm1 ins 362, 367E constructs were generated as described [14], [20]. Site directed mutagenesis was performed by the Quik-Change Site-Directed Mutagenesis Kit (Stratagene, La Jolla, CA).

### Cells and Tissue Culture

Experiments involving the preparation of mouse embryonic fibroblasts (MEFs) from 14 day pregnant females were performed following the guidelines given in Anzeige Nr. 9.93.2.10.44.07.189 to licence Nr: 50.203.2-K 13, 21/02 “Cell-biological basis of innate immunity” issued by LANUV NRW (North Rhine-Westphalia).

C57BL/6 embryonic fibroblasts (MEFs) were prepared from mice at day 14 *post coitum* and cultured in DMEM (high glucose) (Invitrogen, Carlsbad, CA) supplemented with 10% FCS (Biochrom AG, Berlin, Germany), 2 mM L-glutamine, 1 mM sodium pyruvate, non-essential amino acids, 100 U/ml penicillin, 100 mg/ml streptomycin (all PAA, Pasching, Austria). Cells were transiently transfected using FuGENE6 (Roche, Basel, Switzerland) according to the manufacturer's instructions. Mouse IFNγ was purchased from PeproTech, NJ, USA.

### Transferrin Uptake Experiments

Cells were grown on coverslips and starved for 1 hour in FCS-free medium. To label early endosomes, Alexa-546 labeled human Transferrin (Molecular Probes, Carlsbad, CA) was diluted to a final concentration of 5 µg/ml into the FCS-free medium, and cells were incubated at 37°C, 7.5% CO_2_ for 5 minutes. Cells were then fixed with ice-cold 3% PFA in PBS for 20 minutes. To label recycling endosomes, the cells were incubated with FCS-free medium with diluted transferrin for 10 minutes after starvation, then washed 3 times with ice-cold PBS. Complete medium was added afterwards and the cells were incubated at 37°C, 7.5% CO_2_ for 30 minutes. Finally cells were fixed with ice-cold 3% PFA in PBS for 20 minutes followed by three washes with PBS and stained for immunofluorescence.

### Lysotracker Loading Experiments

Lysotracker Red DND-99 (Molecular Probes) was diluted to a final concentration of 50 nM in complete medium and the cells were incubated at 37°C, 7.5% CO_2_ for 20 minutes. Cells were then fixed with ice-cold 3% PFA in PBS for 20 minutes followed by three washes with PBS and stained for immunofluorescence.

### Latex Bead Phagocytosis Experiments

MEFs were grown on the coverslips, treated with 200 U/ml IFNγ and/or transfected with indicated constructs for 24 hours. During the IFNγ-treatment and/or transfection procedure, 2 µm carboxylated latex beads (Polysciences, Warrington, PA) were added to the culture at a dilution of 1∶1000, and the cells were incubated with the latex beads at 37°C overnight. The latex beads were extensively phagocytosed by the MEFs through unidentified receptors. Finally the cells were fixed with 3% PFA for 20 minutes at room temperature and stained for immunofluorescence.

### Peptide-Streptavidin Complexes

The Irgm1 αK peptide H-SK(Biotin)LRLMTCAIVNAFFRLLRFLPCVCC-OH was synthesized by JPT Peptide Technologies GmbH, Berlin.

The peptide was biotinylated on Lysine at the second position of the sequence. The peptide was dissolved in 10 mM NaAc pH 4.5 buffer with 10 mM TCEP as reducing agent and stored at −80°C at a stock concentration of ∼100 µM. The absolute absorbance of the Irgm1 αK peptide at 230 nm in the dissolving buffer was ∼0.46/100 µM.

The peptide-Cy3-streptavidin complex was made in the blocking buffer used for the immunofluorescence staining (PBS pH 7.4/0.1% saponin/1% BSA with additional 2 mM DTT). The Cy3-labelled streptavidin (Sigma, S6402) was first diluted in the blocking buffer to a final concentration of 20 µg/ml (0.33 µM). The peptide stock solution was then added to the streptavidin solution with a titration from 4∶1 to 50∶1 molar ratio (peptide to streptavidin). The optimum ratio was experimentally determined based on the staining signals (8 µM peptide in the Figure 6). The peptide-streptavidin solution was mixed and precipitated material removed by centrifugation at 45.000 rpm for 30 minutes. The supernatant was recovered and antibodies against the indicated proteins added. This solution was used like a primary antibody in immunofluorescence staining, and was incubated with PFA-fixed saponin permeabilized cells at 37°C for 1 hour. The peptide-streptavidin images were obtained in the Cy3 channel.

### Immunofluorescent Staining

Immunofluorescent staining was performed on paraformaldehyde-fixed cells essentially as described earlier [20]. Images were taken with a Zeiss Axioplan II fluorescence microscope equipped with an AxioCam MRm camera (Zeiss) and processed with Axiovision 4.6 software (Zeiss). 4′,6-Diamidine-2′-phenylindole dihydrochloride (DAPI, Invitrogen) was used for nuclear counterstaining at a final concentration of 0.5 µg/ml.

### Immunoreagents

The following serological reagents were used for immunofluorescence at the indicated concentrations: anti-Irgm1 goat polyclonal antibody P20 (1∶100, Santa Cruz Biotechnology), anti-Irgm1 mouse monoclonal antibody 1B2 (supernatant recovered from hybridoma cell culture [24]), anti-Irgm2 rabbit antiserum H53 (1∶1000, [6]), anti-GM130 mouse monoclonal antibody (1∶1000, BD Transduction Lab, 610822), anti-TGN38 goat polyclonal antibody S-20 (1∶100, Santa Cruz Biotechnology), anti-CI-M6PR rabbit antiserum (1∶100, a gift from Albert Haas, University of Bonn), anti-LAMP1 rat monoclonal antibody 1D4B (1∶1000, University of Iowa, USA), goat anti-mouse Alexa 488 and 546, goat anti-rabbit Alexa 488 and 546, donkey anti-rat Alexa 488, donkey anti-goat Alexa 350, 488, 546 and 647, donkey anti-mouse Alexa 488, 555 and 647, donkey anti-rabbit Alexa 488, 555 and 647 (1∶1000, Molecular Probes, Invitrogen).

The specificity of the two anti-Irgm1 reagents used, monoclonal 1B2 and goat serum P20 was confirmed by staining IFNγ-induced MEFs from Irgm1-deficient mice (Figure S2). No staining was detected, compared with strong typical Irgm1 localisation patterns in wild type MEFs stained with the same reagents at the same time.

## Supporting Information

Figure S1 Mislocalisation of Irgm1-EGFP (pF25) tagged construct in RAW264.7 macrophages. RAW 264.7 macrophages were transfected with the pF25 Irgm1-EGFP C-terminal tagged construct as described in Materials and Methods, and induced for 24 h with 100 U IFNγ (panels A) or not induced (panels B). A further set of RAW264.7 macrophages were induced with 100 U IFNγ but not transfected (panels C). After 24 hr, all cells were fixed and stained for the Golgi membrane marker, GM130. Untransfected cells (panels C) were also stained with goat anti Irgm1 serum P20. EGFP and Alexa-488 donkey anti-goat were detected at 488 nm (green), while GM130 was detected with Alexa-555 donkey anti mouse at 555 nm (red). Nuclei were counterstained with DAPI The transfected pF25 construct labelled multiple unidentified cytoplasmic components, but failed to localise to the Golgi as defined by GM130. In contrast, anti-Irgm1 serum P20 clearly co-localised with GM130, with additional weak staining of other cytoplasmic components. In further stainings, transfected pF25 could be partially localised to a LAMP1-positive compartment (not shown).
(4.38 MB TIF)

Figure S2 Specificity controls for goat anti Irgm1 antiserum P20 and mouse anti Irgm1 monoclonal 1B2. The two anti-Irgm1 reagents were used to stain primary MEFs induced for 24 hr with 100 U IFNg, followed by appropriate fluoresceinated secondary anti-immunoglobulin reagents (see Materials and Methods). MEFs were from wild-type C57BL/6 (top row) or from Irgm1-deficient C57BL/6 mice (Collazo et al). See Materials and Methods for further details. For both primary Irgm1-specific reagents typical Golgi and cytoplasmic staining was observed in the wild-type MEFs, while only barely visible cytoplasmic staining was seen in the Irgm1-deficient MEFs.
(4.48 MB TIF)

Table S1 C57BL/6 MEFs were induced for 24 h with IFNγ at 100 U/ml. They were then stained according to Materials and Methods with one or the other of two triple staining protocols, above. The co-localisation of Irgm1 with the standard lysosomal (LAMP-1) marker, or anti- Irgm2 as a Golgi marker (Martens and Howard, 2006 The Interferon-Inducible GTPases. Annu Rev Cell Dev Biol 22: 559–589) was recorded in approximately 50 cells for each protocol. It is clear that weak and strong Golgi staining is randomly associated with weak or strong lysosomal staining.
(0.04 MB DOC)
