# Supplementary figures and images for: Localisation and Mislocalisation of the Interferon-Inducible Immunity-Related GTPase, Irgm1 (LRG-47) in Mouse Cells
Source: PLoS One. 2010 Jan 13;5(1):e8648. doi: 10.1371/journal.pone.0008648 (PMC2799677; doi:10.1371/journal.pone.0008648)

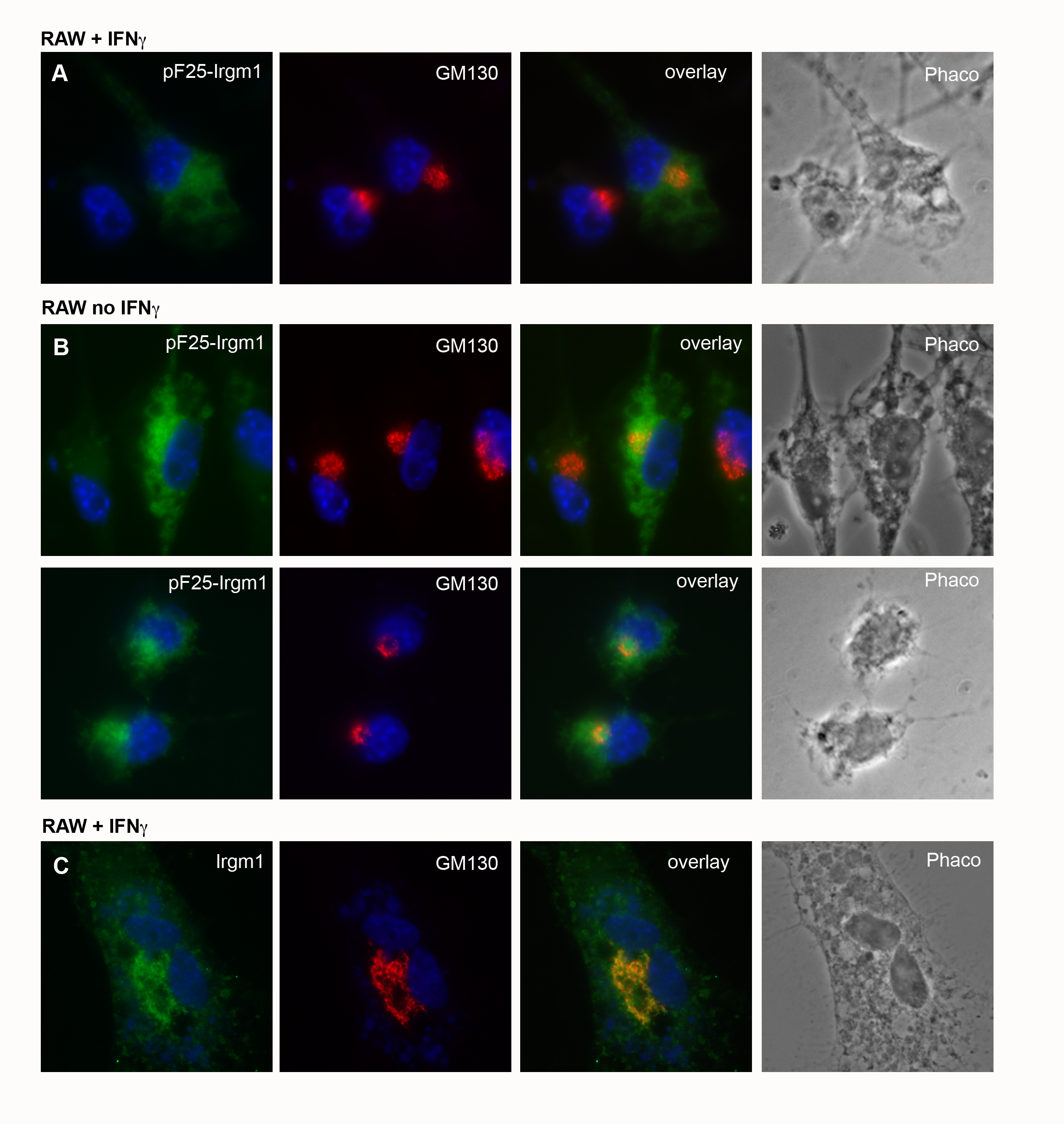

Supplement: Figure S1 — Mislocalisation of Irgm1-EGFP (pF25) tagged construct in RAW264.7 macrophages. RAW 264.7 macrophages were transfected with the pF25 Irgm1-EGFP C-terminal tagged construct as described in Materials and Methods, and induced for 24 h with 100 U IFNγ (panels A) or not induced (panels B). A further set of RAW264.7 macrophages were induced with 100 U IFNγ but not transfected (panels C). After 24 hr, all cells were fixed and stained for the Golgi membrane marker, GM130. Untransfected cells (panels C) were also stained with goat anti Irgm1 serum P20. EGFP and Alexa-488 donkey anti-goat were detected at 488 nm (green), while GM130 was detected with Alexa-555 donkey anti mouse at 555 nm (red). Nuclei were counterstained with DAPI The transfected pF25 construct labelled multiple unidentified cytoplasmic components, but failed to localise to the Golgi as defined by GM130. In contrast, anti-Irgm1 serum P20 clearly co-localised with GM130, with additional weak staining of other cytoplasmic components. In further stainings, transfected pF25 could be partially localised to a LAMP1-positive compartment (not shown). (4.38 MB TIF) [file pone.0008648.s001.tif]

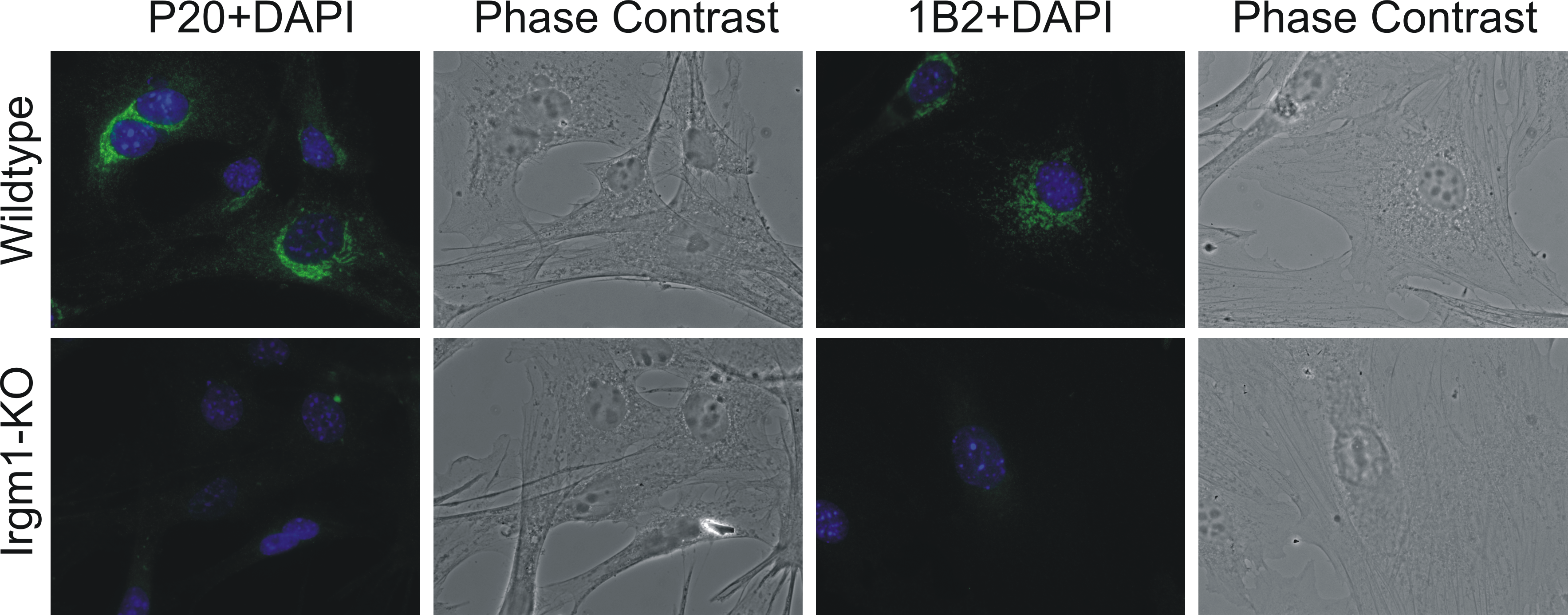

Supplement: Figure S2 — Specificity controls for goat anti Irgm1 antiserum P20 and mouse anti Irgm1 monoclonal 1B2. The two anti-Irgm1 reagents were used to stain primary MEFs induced for 24 hr with 100 U IFNg, followed by appropriate fluoresceinated secondary anti-immunoglobulin reagents (see Materials and Methods). MEFs were from wild-type C57BL/6 (top row) or from Irgm1-deficient C57BL/6 mice (Collazo et al). See Materials and Methods for further details. For both primary Irgm1-specific reagents typical Golgi and cytoplasmic staining was observed in the wild-type MEFs, while only barely visible cytoplasmic staining was seen in the Irgm1-deficient MEFs. (4.48 MB TIF) [file pone.0008648.s002.tif]
