# Supplementary material for: Localisation and Mislocalisation of the Interferon-Inducible Immunity-Related GTPase, Irgm1 (LRG-47) in Mouse Cells
Source: PLoS One. 2010 Jan 13;5(1):e8648. doi: 10.1371/journal.pone.0008648 (PMC2799677; doi:10.1371/journal.pone.0008648)
Supplement: Table S1 — C57BL/6 MEFs were induced for 24 h with IFNγ at 100 U/ml. They were then stained according to Materials and Methods with one or the other of two triple staining protocols, above. The co-localisation of Irgm1 with the standard lysosomal (LAMP-1) marker, or anti- Irgm2 as a Golgi marker (Martens and Howard, 2006 The Interferon-Inducible GTPases. Annu Rev Cell Dev Biol 22: 559–589) was recorded in approximately 50 cells for each protocol. It is clear that weak and strong Golgi staining is randomly associated with weak or strong lysosomal staining. (0.04 MB DOC) [file pone.0008648.s003.doc]

**Table S1**

**Distribution of Irgm1 in IFNg-stimulated MEFs**

Staining:

1) 53 cells evaluated

a-Irgm1(P20) detected with donkey a-goat-546

a-Irgm2(H53) detected with donkey a-rabbit-647

a-LAMP1 (1D4B) detected with donkey a-rat 488

2) 49 cells evaluated

a-irgm1(P20) detected with donkey a-goat-546

a-irgm2(H53) detected with donkey a-rabbit-488

a-LAMP1 (1D4B) detected with donkey a-mouse-647

102 cells evaluated in total (results pooled)

| **Irgm1-Golgi-staininig** | | | |
| --- | --- | --- | --- |
| **weak** | | **strong** | |
| lysosomal association of Irgm1 | | lysosomal association of Irgm1 | |
| weak | strong | weak | strong |
| 27 cells | 23 cells | 22 cells | 30 cells |
| 27% | 23% | 22% | 29% |

**Legend to Table S1**

C57BL/6 MEFs were induced for 24 h with IFNγ at 100 U/ml. They were then stained according to Materials and Methods with one or the other of two triple staining protocols, above. The co-localisation of Irgm1 with the standard lysosomal (LAMP-1) marker, or anti- Irgm2 as a Golgi marker (Martens and Howard, 2006 The Interferon-Inducible GTPases. Annu Rev Cell Dev Biol 22: 559-589) was recorded in approximately 50 cells for each protocol. It is clear that weak and strong Golgi staining is randomly associated with weak or strong lysosomal staining.
